# Loose Coupling between the Voltage Sensor and the Activation Gate in Mammalian HCN Channels Suggests a Gating Mechanism

**DOI:** 10.3390/ijms25084309

**Published:** 2024-04-13

**Authors:** Xiaoan Wu, Kevin P. Cunningham, Andrew Bruening-Wright, Shilpi Pandey, H. Peter Larsson

**Affiliations:** 1 Department of Physiology and Biophysics, Miller School of Medicine, University of Miami, Miami, FL 33136, USA; xxw320@miami.edu (X.W.); k.cunningham@westminster.ac.uk (K.P.C.); 2 School of Life Sciences, University of Westminster, London W1W 6UW, UK; 3 Charles River Laboratories, Cleveland, OH 44128, USA; andrew.bruening-wright@crl.com; 4 Oregan National Primate Research Center, Oregon Health & Science University, Beaverton, OR 97006, USA; pandeys@ohsu.edu; 5 Department of Biomedical and Clinical Sciences, Linköping University, 58185 Linköping, Sweden

**Keywords:** HCN channels, Kv channels, voltage clamp fluorometry, voltage gating, electromechanical coupling

## Abstract

Voltage-gated potassium (Kv) channels and hyperpolarization-activated cyclic nucleotide-gated (HCN) channels share similar structures but have opposite gating polarity. Kv channels have a strong coupling (>10^9^) between the voltage sensor (S4) and the activation gate: when S4s are activated, the gate is open to >80% but, when S4s are deactivated, the gate is open <10^−9^ of the time. Using noise analysis, we show that the coupling between S4 and the gate is <200 in HCN channels. In addition, using voltage clamp fluorometry, locking the gate open in a Kv channel drastically altered the energetics of S4 movement. In contrast, locking the gate open or decreasing the coupling between S4 and the gate in HCN channels had only minor effects on the energetics of S4 movement, consistent with a weak coupling between S4 and the gate. We propose that this loose coupling is a prerequisite for the reversed voltage gating in HCN channels.

## 1. Introduction

Hyperpolarization-activated cyclic nucleotide-gated (HCN) channels are mainly activated by membrane hyperpolarization and play important roles in pacemaking, dendritic integration, resting membrane potentials, and synaptic transmission [1]. Removal of the currents through HCN channels, by either knock-out of HCN channels in mice or by naturally occurring mutations of HCN channels in humans, results in heart arrhythmia, epilepsy, and altered spatial memory, further showing the physiological importance of HCN channels [2,3].

Both the hyperpolarization-activated HCN channels and the depolarization-activated potassium (Kv) channels belong to the superfamily of voltage-gated ion channels [4,5]. HCN and Kv channels share many common structural features. For example, these channels comprise four subunits, each with six transmembrane domains and a pore loop [6,7,8]. The fourth transmembrane domain, S4, has a unique sequence with positively charged amino acids at every third position in both HCN and Kv channels. Both channels use their positively charged S4 as their main voltage sensor [9]. The movement of S4 is conserved between the two types of channels: in response to depolarization, S4 charges move toward the extracellular solution and, in response to hyperpolarization, S4 charges move toward the intracellular solution [10,11,12]. In addition, both channels use the intracellular end of the sixth transmembrane domain (S6) as the activation gate that closes off the access for ions to the pore at the intracellular end of the pore. Therefore, S4 movements induce the opening and closing of the gate through voltage-sensor-to-gate coupling, known as electromechanical coupling [9]. In response to hyperpolarization, Kv channels close their gate in response to the inward S4 movement [13,14], while HCN channels open their activation gate in response to the inward S4 movement [15,16,17,18,19]. The mechanism for this reversed voltage gating in HCN channels compared to in Kv channels is not completely understood.

Both crystallographic and mutagenesis data have suggested that the S4–S5 linker couples S4 movement to the activation gate in depolarization-activated K channels [13,20,21,22]. For example, chimeric channels between KcsA and Shaker K channels were opened by depolarization only when the chimeric channels contained both the S4–S5 linker and lower S6 from the depolarization-activated Shaker K channel [21]. Direct interactions between S4–S5 linker and residues in the lower S6 shown in the crystal structure of Kv1.2 channels were used to propose a model for how the S4–S5 linker couples to the S6 gate in Kv channels: outward movement of S4 moves the S4–S5 linker outwards, pulling the S6 gate open; inward movement of S4 moves the S4–S5 linker inwards, pushing the S6 gate shut [20]. However, the S4–S5 linker has been shown to not be necessary for HCN gating as the lack of the S4–S5 linker does not prevent gating in HCN channels [23], which suggests a different voltage sensor-to-gate coupling in these channels.

Recent cryo-EM studies have revealed a structural difference between most Kv and HCN channels: most Kv channels display domain-swapped structures, while HCN channels display non-domain-swapped structures [6]. That is to say, the voltage-sensing domain (VSD) interacts with the pore domain (PD) in the same subunit in HCN channels, while, in most Kv channels, the VSD interacts with the PD between two neighboring subunits. However, this difference is not the mechanism of reversed voltage sensor-to-gate coupling between HCN channels and Kv channels, as two depolarization-activated Kv channels, EAG and hERG [8,24], are also non-domain-swapped.

Several lines of evidence suggest that the coupling between S4 and the activation gate is very strong in domain-swapped Kv channels. (1) The minimum open probability at negative potentials is very low (<10^−9^) in Shaker channels [25], suggesting that the opening of the activation gate is very unlikely to occur in channels with S4 in the resting state. (2) The maximum open probability is very high (>80%), suggesting that the outward movement of S4 drastically increases (>10^9^ fold) the stability of the open state [25]. (3) The movement of S4 charges, which gives rise to the gating currents, is drastically altered in open Shaker K channels, so that the off-gating currents are slowed in open channels [26,27]. This is possibly because the gating charges cannot return to the resting state before the channels have closed, suggesting a tight coupling between S4 movement and the gate position. On the other hand, non-domain-swapped channels might have a weak coupling, as a previous spHCN study suggested a weak coupling of voltage sensors and gate in sea urchin spHCN channels by measuring the charge movement in gating-locked spHCN channels [28].

We tested the coupling between S4 and the activation gate in HCN channels using noise analysis and voltage clamp fluorometry. We show that HCN channels display a much smaller increase in the open probability between HCN channels with all S4s in the resting state and all S4s in the activated state compared to in Kv channels. This suggests that HCN channels have a much looser coupling between S4 and the gate compared to Kv channels. To further support this conclusion, we found that the S4 movement was not altered drastically by mutations that either locked HCN channels open or decreased the coupling between S4 and the gate in HCN channels. This contrasts with Kv channels, which showed large changes in the S4 movement for Kv channels that were locked open. We propose that the loose coupling in HCN channels is a prerequisite for the reversed voltage gating.

## 2. Results

### 2.1. Weak Coupling in HCN Channels

**Figure 1:**
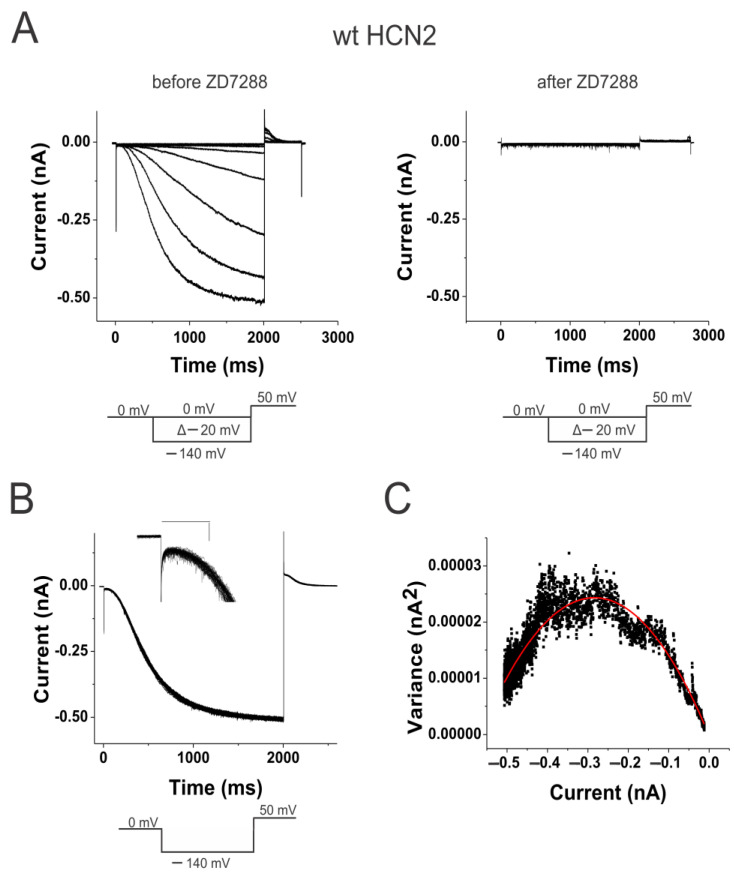
Minimum and maximum open probabilities indicate a loose coupling in wt HCN2 channels. (**A**) wt HCN2 currents (left) before and (right) after 100 µM ZD7288 in response to voltage steps between 0 mV and −140 mV from a holding potential of 0 mV with a tail potential of +50 mV. (**B**) Currents in response to 100 sweeps to −140 mV. (insert) Enlargement of the initial 200 ms of the 100 sweeps, showing the amplitude of the initial currents. Scale bar 100 ms, 10 pA. (**C**) Variance-to-mean plot of data from (**B**). Data fitted with the equation var(I) = I(i − I/N). Single-channel current i = 0.17 pA. Number of channels N = 3288. Minimum open probability = 0.02 and maximum open probability = 0.89, in this patch.

**Figure 2:**
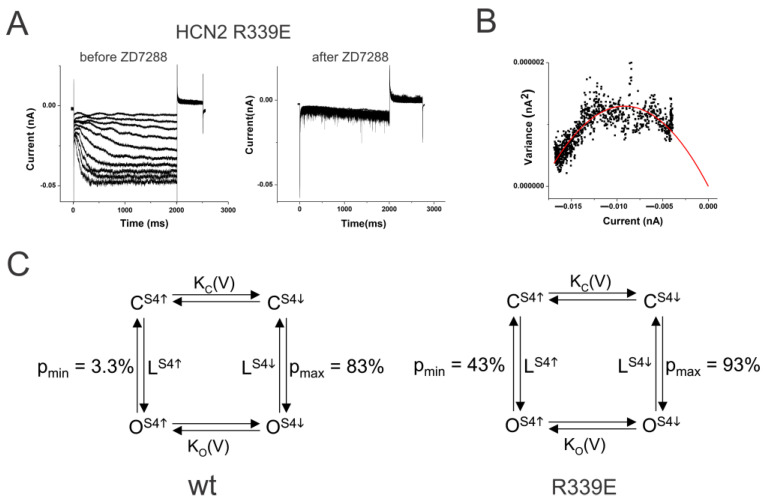
The mutation R339E makes the coupling looser. (**A**) HCN2 R339E currents (left) before and (right) after 100 µM ZD7288 in response to voltage steps in Figure 1A. (**B**) Variance-to-mean plot of R339E currents fitted with the equation var(I) = I(i − I/N). Single-channel current i = 0.19 pA. Number of channels N = 174. Minimum open probability = 0.32 and maximum open probability = 0.91 in this patch. (**C**) Simple four-state model for (left) wt HCN2 channels and (right) HCN2 R339E channels. LS4^↑^ and LS4^↓^ are the equilibrium constants for the open transition with S4 resting and activated, respectively. pmin and pmax are the open probabilities with S4 resting and activated, respectively.

We expressed wild-type (WT) mouse HCN2 channels in *Xenopus* oocytes and measured the currents in cell-attached patches under voltage clamp (Figure 1A). Using non-stationary noise analysis, we measured the change in open probability at a membrane potential (0 mV) that keeps the channels maximally closed and then stepped to a membrane potential (−140 mV) that opens the channels maximally (Figure 1B). The currents were measured in 100 consecutive steps, and the average and variance from these traces were calculated (Figure 1C). We applied 100 µM of the HCN-channel-specific blocker ZD7288 to estimate the background current and variance. The background current and variance were subtracted off-line from the variance-to-mean curves of the HCN currents. By fitting the variance-to-mean data to a parabola, we estimated the minimum and maximum open probability at the beginning and end of the voltage step. The minimum open probability (p_min_) is assumed to be the open probability when no S4 has activated (S4^up^), while the maximum open probability (p_max_) is assumed to be the open probability when all four S4s have activated (S4^down^) (Figure 2C). For WT HCN2 channels, we found that p_min_ was 3.3% ± 1.3% (*n* = 6) and p_max_ was 82.7% ± 3.7% (*n* = 6). These two open probabilities are converted to two equilibrium constants, L_S4_^up^ and L_S4_^down^, for the opening gate in channels with all S4s at rest and all S4s activated, respectively (see Section 4.5: L_S4_^up^ = (p_min_^−1^ − 1)^−1^ and L_S4_^down^ = (p_max_^−1^ − 1)^−1^. The ratio of these two equilibrium constants, L = L_S4_^down^/L_S4_^up^, gives the coupling factor between S4 position and the opening of the activation gate for a simple channel model. In WT HCN2 channels, the coupling factor was 140. This corresponds to a coupling energy of 2.9 kcal/mole (∆∆G =RT ln (140) = 2.9 kcal/mole), which is similar to a coupling energy of 3~4.5 kcal/mole in sea urchin spHCN channels measured by charge movement [28]. The coupling energy reflects the relative stability imparted to the open state when S4 is in the down position, compared to when S4 is in the up position.

### 2.2. S4–S5 Mutation Further Weakens the Coupling

We then measured the coupling factor in HCN2 R339E mutant channels. Mutation R339E has been proposed to decrease the coupling between S4 and the gate in HCN2 channels [29]. The R339E mutation displayed a large instantaneous leak current in response to hyperpolarization (Figure 2A). This leak current was blocked by the application of 100 µM ZD7288, showing that these currents are from HCN channels. In addition, the minimum open probability of R339E was 43.3% ± 13% (*n* = 4) and the maximum open probability (p_max_) was 93.1% ± 2.4% (*n* = 4) (Figure 2B). The ratio of the equilibrium constants, L = L_S4_^down^/L_S4_^up^, in R339E mutation was 17.6 compared to 140 in WT HCN2 channels (Figure 2C). This corresponds to a change in the coupling energy by ∆∆G = RT ln (140/17.6) = 1.2 kcal/mole. A lower coupling factor between S4 position and the opening of the activation gate in R339E channels than in WT HCN2 channels shows that the mutation R339E reduces the coupling between S4 and the gate. A decrease in the coupling energy by only 1.2 kcal resulted in a drastic change in the minimum open probability, which further shows that HCN2 channels have a loose coupling that can easily be disturbed.

### 2.3. Reciprocal Effects of Gate and Voltage Sensor in Strongly Coupled Ion Channels

An allosteric mechanism of the S4-to-gate coupling is that the coupling is bidirectional: the position of S4 affects the movement of the gate but, also, the position of the gate will affect the movement of S4. For a strongly coupled channel, such as the Shaker K channel [25], the movement of S4 should be drastically altered by the position of the gate. On the other hand, for a weakly coupled channel, such as the HCN channel, the movement of S4 should be much less influenced by the position of the gate. To test these assumptions, we measured the S4 movement and the opening of the gate simultaneously by using VCF on fluorescent-labeled, conducting Shaker K channels and HCN channels [30]. In addition, we introduced mutations that allowed us to chemically lock these channels open and tested how it would affect the S4 movements.

**Figure 3:**
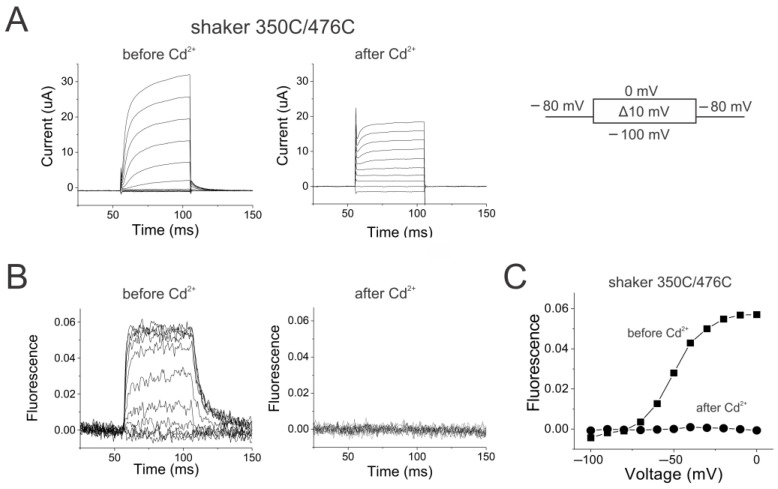
Locking the activation gate open in Shaker channels prevents S4 movement. (**A**) Shaker 350C/476C currents (left) before and (right) after injection of the oocyte with 50 nL of 10 µM CdCl_2_ in response to voltage steps from −100 mV to 0 mV from a holding potential of −80 mV with a tail potential of −80 mV. (**B**) Shaker 350C/476C fluorescence (left) before and (right) after injection of the oocyte with 50 nL of 10 mM CdCl_2_ in response to voltage steps in Figure 3A. (**C**) FV relations before (squares) and after (circles) locking the channels open by Cd^2+^ application on shaker 350C/476C channels.

We measured S4 movement in Shaker K channels by introducing a cysteine at position 350 and labeling this cysteine with Alexa-488-maleimide. Changes in the fluorescence from fluorophores attached to 350C have previously been shown to report on S4 movement in Shaker K channels [31,32]. To lock the Shaker channel open, we introduced an additional cysteine at position 476 in S6. When Cd^2+^ binds to 476C in Shaker channels, 476C in one subunit co-ordinates a Cd^2+^ with H486 from a different subunit and locks the channels in their open state [33]. Figure 3 shows the currents and the fluorescence recorded simultaneously from Alexa488-labeled 350C/476C Shaker channels, both of which are similar to those from Alexa488-labeled 350C channels as previously reported [31]. This shows that the 476C mutation does not alter the channel function before the application of Cd^2+^.

After the application of Cd^2+^, the currents became largely ohmic, accompanied by the removal of tail currents, suggesting that the channels were locked in the open state (Figure 3A). A small fraction of the voltage-dependent Shaker currents remained, which could be due to some channels located far from the Cd^2+^ injection site. The fraction of voltage-dependent currents decreased over time, as diffusion of Cd^2+^ inside the oocyte is reaching more and more channels. Note that the currents are measured from the whole oocyte, while the fluorescence is measured from a smaller area close to the Cd^2+^ injection site. The fluorescence changes disappeared with Cd^2+^ application, suggesting that S4 is locked in these channels (Figure 3B). For an allosteric mechanism with a strong coupling between S4 and the gate, one expects the locking of the activation gate to have a profound effect on the ability of S4 to move. This effect is clearly seen in the FV relation shown in Figure 3C—after locking the channel open, there is no detectable S4 movement.

### 2.4. Reciprocal Effects of Gate and Voltage Sensor in Weakly Coupled Ion Channels

To test whether S4 in HCN channels would be similarly affected by locking the gate open, we measured S4 movement in spHCN channels by introducing a cysteine at position R332 and labeled this cysteine with Alexa488-maleimide. The changes in fluorescence from Alexa488 attached to 332C have previously been shown to report on S4 movement in spHCN channels [17,30]. To lock the channel gate open, we introduced two additional cysteines in S6, H462C, and L466C into the Alexa-488-labeled 332C spHCN channels. 462C in one subunit co-ordinates a Cd^2+^ with 466C from the same subunit and locks the channels in the open state [34]. Cd^2+^ locks the channel gate in the open state most likely by crosslinking the two cysteines in one S6 (Figure S1), thereby stabilizing a bend in the open conformation of S6 (Movie S1) [6,19].

**Figure 4:**
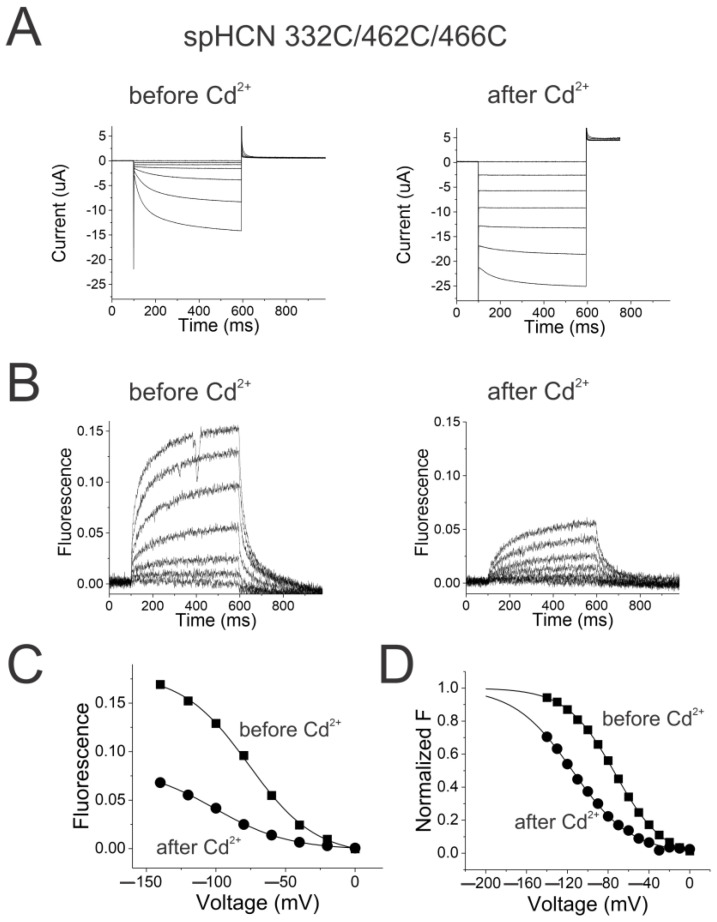
Locking the activation gate open in spHCN channels does not prevent S4 movement. (**A**) SpHCN 332C/462C/466C currents before (left) and after (right) injection of the oocyte with 50 nL of 10 µM CdCl_2_ in response to voltage steps in Figure 1A. (**B**) SpHCN 332C/462C/466C fluorescence before (left) and after (right) injection of the oocyte with 50 nL of 10 µM CdCl_2_ in response to voltage steps in Figure 1A. (**C**) FV relations before (squares) and after (circles) locking the channels open by Cd^2+^ application on spHCN 332C/462C/466C channels. (**D**) Normalized FV relations from (**C**).

The currents and the fluorescence from 332C/462C/466C spHCN channels are similar to those from 332C channels (cf. Figure 4A and Figure S2A), showing that the 462C/466C mutations do not alter drastically the channel function before the application of Cd^2+^. The application of Cd^2+^ converts the spHCN currents into largely ohmic currents, suggesting that the 332C/462C/466C spHCN channels are locked in the open state (Figure 4A), which is consistent with previous studies [28,34]. As a control, Cd^2+^ application on 332C channels did not result in a locked open phenotype (Figure S2A). More importantly, the detectable fluorescence changes from 332C/462C/466C spHCN channels after Cd^2+^ application suggest that S4 still moves when spHCN channels are locked open (Figure 4B).

The amplitude of the fluorescence signal from 332C/462C/466C spHCN channels decreased over time (Figure 4C). A similar decrease was seen in 332C channels (Figure S2C). This decrease is most likely due to the internalization of fluorescently labeled channels and bleaching of the Alexa fluorophores compared to the background fluorescence. In addition, the voltage dependence of the fluorescence signal in 332C/462C/466C spHCN channels before Cd^2+^ injection was −65.0 ± 3.3 mV (*n* = 3) versus −91.0 ± 4.1 mV (*n* = 3) after Cd^2+^ injection. Thus, locking the channels open shifts the FV by only −25 mV (Figure 4D). Cd^2+^ application on 332C channels causes similar voltage shifts in the FV relation (Figure S2D): from −51.2 ± 1.5 mV (*n* = 6) to −71.0 ± 3.8 mV (*n* = 6). The similar voltage shift in both cases is probably due to the screening of intracellular surface charges by Cd^2+^.

For an allosteric mechanism with a weak coupling between S4 and the gate, one expects the locking of the activation gate to have only a small effect on the ability of S4 to move. Because locking the channel gate open had only a small effect on the fluorescence signal, we conclude that S4 is not strongly coupled to the gate in HCN channels.

### 2.5. Coupling Mutation Affects Both Open Probability and S4 Movement

We used a simple allosteric model for HCN channels (Figure S3A) based on a previous model [35] to predict the effect of the HCN2 R339E mutation on S4 movement in channels with different coupling factors. The drastic increase in open probability at positive potentials caused by the R339E mutation could be due to a destabilization of the closed state with all S4s up (C0) or due to a stabilization of the open state with all S4s up (O0) (Figure S3A). If HCN channels had a strong coupling, a destabilization of C0 or a stabilization of O0 would shift the S4 movement dramatically by affecting the K_C_ or K_O_ equilibrium constants, respectively. For example, if HCN channels had a strong coupling factor of 10^9^, as shown for Shaker K channels [25], and the R339E-caused increase in the open probability is due to a destabilization of C0, then this would induce a voltage shift of the midpoint of S4 movement by >−120 mV (Figure S3B). Alternatively, if the R339E-caused increase in the open probability is due to a stabilization of O0, then this would induce a voltage shift of the midpoint of the “locked-open” S4 movement by >120 mV (Figure S3C). We tested these hypotheses in spHCN channels using voltage clamp fluorometry (VCF).

**Figure 5:**
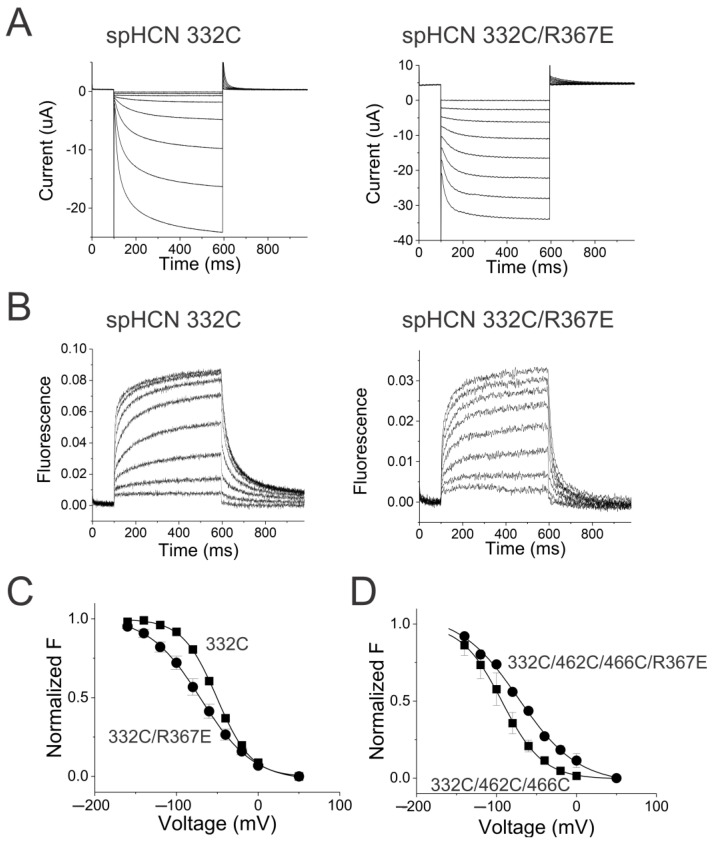
The decoupling mutation R367E has only small effects on S4 movement in spHCN channels. (**A**) Currents of spHCN 332C (left) and 332C/R367E (right) in response to voltage steps in Figure 1A. (**B**) Fluorescence of spHCN 332C (left) and 332C/R367E (right) in response to voltage steps in Figure 1A. (**C**) Normalized FV relations of 332C (squares) and 332C/R367E (circles). (**D**) Normalized “locked-open” FV relations of 332C/462C/466C (squares) and 332C/462C/466C/R367E (circles) in the presence of Cd^2+^, measured as in Figure 4D.

We first tested the effect of the mutation R367E (homologous to mutation R339E in HCN2 channels) on the S4 movement in spHCN channels. Similar to the R339E mutation in HCN2 channels, the R367E mutation in spHCN channels made the channels leaky with large ohmic currents at depolarized voltages, suggesting that these channels do not close their activation gate at depolarized potentials (Figure 5A). These channels are still gated by voltage as seen by the increase in the currents at hyperpolarized voltages. R367E did not dramatically shift the FV relation of 332C channels (Figure 5C). The midpoint of the FV curve from 332C/R367E channels was −68.0 ± 5.0 mV (*n* = 6) compared to −52.2 ± 1.5 mV (*n* = 6) for 332C (Figure 5C). We next measured the effect of the R367E mutation on the “locked-open” S4 movement in experiments on Cd^2+^ bound 332C/462C/466C channels (Figure 5D). R367E did not dramatically shift the FV relation of 332C/462C/466C channels. The midpoint of the FV curve from 332C/R367E/462C/466C channels was −73.0 ± 4.2 mV (*n* = 5) compared to −91.0 ± 4.1 mV (*n* = 3) for 332C/462C/466C channels (Figure 5D).

In conclusion, in neither case did the R367E mutation shift the voltage dependence of S4 movement as far as would be predicted if spHCN channels had a strong coupling. The R367E mutation drastically altered the minimum open probability without drastically shifting the voltage dependence of S4 movement, further supporting our conclusion that there is a loose coupling between S4 and the activation gate in HCN channels.

## 3. Discussion

We have shown here that HCN channels have a much looser coupling than Kv channels by both noise analysis and VCF. HCN2 channels have a coupling factor of <200, while Shaker K channels have been shown to have a coupling >10^9^ [25]. Below, we will argue that this looser coupling of HCN channels is one of the factors that allows for the inverted voltage gating in HCN channels. Our data show that S4 movement stabilizes the gate in the closed state compared to the open state by a coupling energy of 2.9 kcal/mole in mammalian HCN2 channels, similar to 3~4.5 kcal/mole in sea urchin spHCN channels [28], and that the mutation R339E in the S4–S5 linker decreases this coupling energy by 1.2 kcal/mole.

A major question in the field of voltage-gated ion channels is how can two related families of ion channels with homologous sequences (HCN and Kv channels) be gated by opposite voltages [36]? Channels in both families have been shown to use S4 as their main voltage sensor, whose positive charges move outwards in response to depolarization and inwards in response to hyperpolarization [11,12,37]. Channels in both families have also been suggested to use an intracellular gate composed of S6 [38,39,40]. Differences in the coupling between S4 and the activation gate have been suggested as the cause for the opposite voltage dependence of HCN channels relative Kv channels [11]. However, how S4 couples to the gate is still not fully clear in either channel family [36].

**Figure 6:**
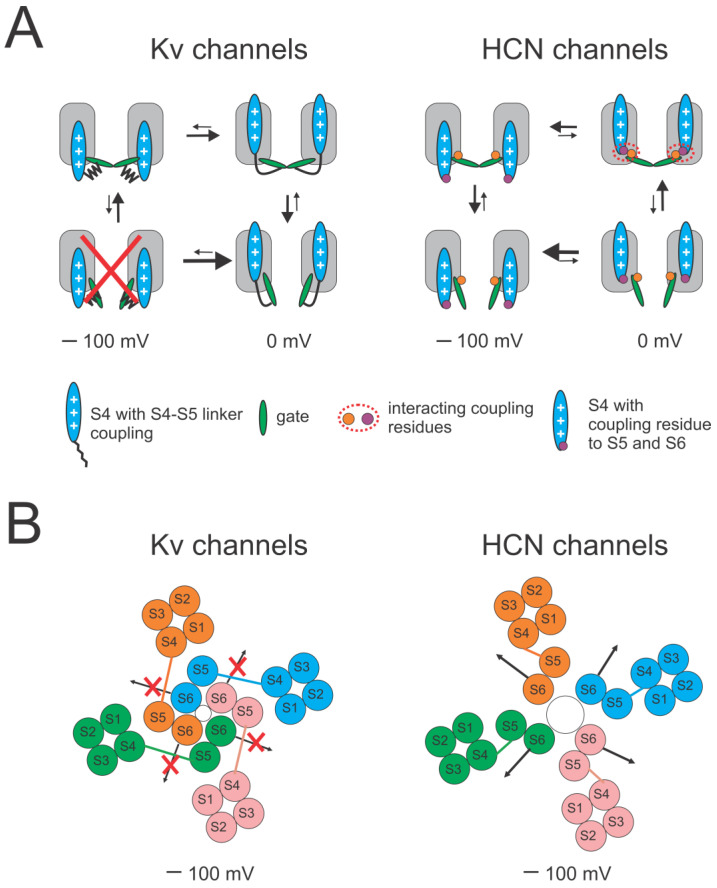
Loose coupling suggests a mechanism for reversed voltage gating. (**A**) A simplified 4-state model for voltage activation of Kv channels and HCN channels with S4 movement (horizontal transitions) between closed and open states (vertical transitions). Only two subunits are shown for clarity. Left: in Kv channels, the steric hindrance between the long S4–S5 linker and S6 prevents both S4 to move down in open Kv channels and the gate to open in Kv channels with S4 down. At 0 mV, outward S4 movement promotes gate opening by pulling S4–S5 linker away from S6, thereby allowing the gate to open. At −100 mV, downward S4 movement stabilizes the closed gate by pushing S4–S5 linker inward and toward the gate, thereby not allowing the gate to open. Red cross indicates Kv channels are not allowed to open when S4 moves down. Right: in HCN channels, the loose coupling between S4 and gate is mainly formed by noncovalent interactions. At 0 mV, outward S4 movement closes the gate by forming interactions between S4 and gate. At −100 mV, downward S4 movement promotes gate opening by removing these interactions. The gate can open when S4 is both outward and inward, and S4 can move in both closed and open channels. The coupling interactions (red dashed circle) stabilize the channels closed when S4 is outward, which explains the lower open probability when S4 is outward than when S4 is inward. (**B**) Top view of models for domain-swapped Kv channels and non-domain-swapped HCN channels upon hyperpolarization. Left: in Kv channels, the long S4–S5 linker from one subunit prevents S6 from the neighboring subunit from moving to open the gate (indicated by red crosses). Right: in HCN channels, the short S4–S5 linker within the same subunit is not limiting the movement of S6, which allows the gate to open. Both Kv and HCN channels are tetrameric with each subunit (labeled pink, blue, orange, and blue) comprising 6 transmembrane segments (S1–S6).

In the model for domain-swapped Kv channels, the S4–S5 linker interacts with S6 in both the closed and open states, so that S4 pulls or pushes on S6 via the S4–S5 linker (Figure 6A, left). At negative voltages, S4 is in the inward state, which positions the S4–S5 linker against the S6 from the neighboring subunit and, therefore, prevents the gate from opening (Figure 6B, left). In response to a depolarization, the outward movement of S4 pulls the S4–S5 linker away from S6 and allows the gate to open. In open Kv channels, S6 pushes against the S4–S5 linker, which keeps S4 in the outward state, thereby preventing inward S4 movement as long as the gate is open. The steric clashes here—the S4–S5 linker preventing the S6 gate from opening at negative voltages and S6 preventing both the S4–S5 linker and S4 from moving inward in open channels—contribute to the strong coupling in domain-swapped Kv channels.

In contrast, our results show that there is a much weaker coupling between S4 and the gate in mammalian HCN2 channels. This weak coupling has also been previously reported in sea urchin spHCN channels [28]. As a non-domain-swapped channel, the HCN channel displays a shorter S4–S5 linker [6] and has been shown to open and close fairly normally even in the absence of the S4–S5 linker [23]. We therefore propose the following model for the reversed voltage gating in HCN channels (Figure 6A, right) with a loose coupling formed by noncovalent interactions between S4 and the pore/gate (S5–S6) [41,42,43] rather than a coupling between the S4–S5 linker and S6: (1) at positive voltages, when S4 is in the outward state, the interactions between S4 and pore/gate (S5–S6) hold the gate closed, as previously suggested [23,42]. (2) In response to a hyperpolarization, the inward movement of S4 breaks these weak closed-state interactions. This allows S6 to move and, thus, the gate to open without steric restrictions by a long S4–S5 linker as in Kv channels (Figure 6B, right). Our model allows for S4 movement in both wt and locked-open HCN channels, as shown in our VCF data. In contrast, our VCF shows no S4 movement in locked-open Shaker channels, probably due to the strong coupling in these channels. In addition, our model allows for gate opening in HCN channels with S4 in both the outward and inward states but with a higher open probability when S4 is inward due to an allosteric coupling between S4 and the gate.

In our models for Kv and HCN channels, similar movements of the voltage sensor S4 and the S6 activation gate occur in both channels but the relative movements of S4 and S6 are opposite in the two channels (Figure 6). A weaker interaction between the S4 and S5–S6 in HCN channels allows S4 to move independently of S5–S6, although they are allosterically coupled [35,44,45]. The interactions are weak in both states, allowing for separation and independent movements of the two regions and then the reformation of a different interaction between the S4 and S5–S6 in the other state. It is the possibility to form different, energetically favorable, open- or closed-state interactions between the S4 and S5–S6 that bias the channels towards the open state or the closed state at hyperpolarized or depolarized voltages, respectively. This model is different from the obligatory coupling proposed for voltage activation of Kv channels, in which the interaction is assumed to be strong, which moves the S4–S5 linker and S6 together during the movement of S4 [25]. We, therefore, suggest that the weaker coupling is part of the answer to why HCN channels have an opposite voltage dependence.

HCN channels display the following features that can be explained by a loose coupling between S4 and the gate: (1) the minimum open probability is large (>10^−2^) in HCN channels. In contrast, Shaker channels have a very low open probability (<10^−9^) with S4 in its down and resting state due to the strong coupling in Shaker channels that does not allow for the gate to open when S4 is in the resting state. (2) Gating currents occur both in open and closed HCN channels, since S4 can move in both closed and open HCN channels [28,45]. In contrast, the strong coupling in Shaker channels does not allow S4 to return to its resting state until the channel gate is closed. This gives rise to a slowing of the off-gating currents and a hook in the gating currents when the channel returns to the resting state after being depolarized to potentials at which the channel opens. (3) HCN channels both open and close with sigmoidal kinetics, since the four S4s can move in both open and closed HCN channels [45,46,47]. In contrast, Shaker K channels open with sigmoidal activation kinetics but close with an exponential time course. In most models of Shaker K channels, this is due to the strong coupling allowing the gate to open with a high probability only from the state with all four S4s activated. (4) Our recent work identified an energetic interaction between the cytosolic side of S4 and S5 that keeps the channel closed when S4 is in the up state [42]. Swapping two residues turned spHCN channels into depolarization-activated channels, further suggesting a weak coupling in HCN channels. Some other non-domain-swapped channels, such as EAG [8] and hERG [24] channels, have also been shown to be able to be re-engineered into channels with opposite voltage dependence by a few mutations [22,48]. We propose that this is because these non-domain-swapped channels do not have the steric clash between a long S4–S5 linker and S6, leading to a loose coupling that can easily be switched into the opposite voltage dependence. However, in domain-swapped Kv channels, no studies on turning them into hyperpolarization-activated have been reported, which is probably due to a much stronger coupling. All these features are consistent with a loose coupling in HCN channels, which contributes to the hyperpolarization-activated mechanism.

Small errors in estimating the minimal open probability and flickering that reduces the maximum open probability might lead to large errors in estimating the coupling factors from open probabilities. In addition, we here assume that the fluorescence reliably reports on the gating charge movement. However, since both measurements of the open probability and the fluorescence suggest a loose coupling in HCN channels, we are confident to conclude that HCN channels have a much looser coupling than Shaker K^+^ channels.

## 4. Materials and Methods

### 4.1. Expression System

Experiments were performed on sea urchin spHCN channels (provided by U.B. Kaupp, Forschungszentrum Julich, Julich, Germany) and mouse HCN2 channels (provided by M.C. Sanguinetti, University of Utah) heterologously expressed in *Xenopus* oocytes. Experiments were carried out according to the guidelines of the Institutional Animal Care & Use Committee at OHSU. The noise analysis experiments were conducted on HCN2 due to their large single-channel conductance compared to spHCN channels. The VCF experiments were conducted on spHCN channels, for which we have previously shown that the fluorescence from fluorescently labeled 332C correlates with the gating currents [30]. In contrast, no gating currents have been reported for HCN2 channels. The HCN2 channel had GC-rich domains in the N-terminus deleted to facilitate the use of molecular biology techniques on the HCN2 DNA [29]. Site-directed mutagenesis was performed on spHCN cDNA (in the pGEM-HE expression vector) using the QuikChange system (Stratagene, San Diego, CA, USA). Single and double mutations in HCN2 cDNA were provided by Dr. Sanguinetti (University of Utah). Some HCN2 mutations were obtained by digesting cDNA from existing mutations and ligating together the appropriate DNA pieces. The presence of the mutations was verified by sequencing. The DNA was linearized with Nhe1 prior to RNA synthesis and purification using a T7 mMessage mMachine kit (Ambion, Austin, TX, USA). A total of 50 nL cRNA (ranging from 2.5 to 50 ng) was injected into each oocyte, and recordings were made by two-electrode voltage clamp (TEVC) 2 to 5 days after injection.

### 4.2. Two-Electrode Voltage Clamp

Whole-cell currents were measured with the two-electrode voltage clamp (TEVC) technique using an Axon Geneclamp 500B (Axon Instruments, San Jose, CA, USA). Microelectrodes were pulled using borosilicate glass and filled with 3M KCl. Each microelectrode had a resistance of 0.5~1.5 MΩ. The bath solution contained, in mM, 89 KCl, 10 HEPES, 0.4 CaCl2, and 0.8 MgCl2 and was adjusted with a pH of 7.4 by KOH. Data were digitized at 5 kHz (Axon Digidata 1322A), filtered at 1 kHz, and monitored and collected using pClamp 10.7 software (Axon Instruments, San Jose, CA, USA).

### 4.3. Voltage Clamp Fluorometry

For voltage clamp fluorometry (VCF) experiments, a single cysteine was introduced in the extracellular side of S4 of spHCN channels. S4 movement in Shaker K channels was measured by introducing a cysteine at position 350 and labeling this cysteine with Alexa-488-maleimide. Oocytes expressing spHCN channels were incubated for 30 min with 100 µM Alexa-488 C5-maleimide (Molecular Probes). After washout, fluorescent-labeled oocytes were placed animal-pole “up” in a bath housed on a Leica DMLFS upright fluorescence microscope. The light was focused on the animal pole of the oocyte through a 20x objective and was passed through a filter cube from Chroma 41,026 (HQ495/30x, Q515LP, HQ545/50 m). Using VCF, the current and the fluorescence were recorded simultaneously. Fluorescence signals were low-pass Bessel filtered (Frequency Devices) at 200 Hz and digitized at 1 kHz. VCF has previously been shown to detect S4 movement in both Shaker K channels and spHCN channels [30,31,43,49,50,51]. For cadmium experiments, 50 nL of 10 µM CdCl_2_ was injected into voltage-clamped oocytes using a Drummond “Nanoject II” nanoliter injector (Drummond Scientific Co., Broomall, PA, USA).

### 4.4. Noise Analysis

Currents in response to 100 sweeps to −120 (or −140) mV were recorded in cell-attached mode using an Axon 200B amplifier and pClamp8 (Axon Instruments Inc., San Jose, CA, USA). Micropatches were recorded with 0.5 MΩ pipettes containing (in mM) 100 KCl, 5 NaCl, 10 HEPES (pH = 7.4), 1 MgCl_2_, 1 CaCl_2_, 0.1 Lanthanum, and 0.1 Gadolinium. Non-stationary noise analyses [52] were calculated from the 100 sweeps using an in-house noise analysis program. Variances were calculated from the ensemble average of the squared differences in successive sweeps, δξ(t), to minimize errors due to drift or instability of the patch [53,54]. Traces with excess noise due to, for example, large, endogenous single-channel currents were excluded if |δξ(t)| > 7[γ(V − V_rev_)I + variance_background_]^1/2^, where γ is the estimated single-channel conductance [54]. Variance-to-mean curves were plotted and fitted with the standard parabolic equation var(I) = Ni^2^p(1 − p) = I(i − I/N) [52]. Control variance and mean currents were measured similarly in the presence of 100 µM of the HCN-channel-specific blocker ZD7288 and subtracted from the variance-to-mean data recorded in the absence of ZD7288 from the same patch. Minimum and maximum open probabilities were obtained from the fit of the variance to mean data from each patch and averaged.

### 4.5. Coupling Constants

At very negative potentials, all S4s are assumed to be in the activated state and the maximum open probability, p_max_, is p_max_ = 1/(1 + L_S4_^down^) (Figure 2C). The opening transition for HCN channels has previously been shown to be voltage-independent [44] and, therefore, L_S4_^down^ is assumed to be voltage-independent. At positive potentials, all S4s are assumed to be in the resting state and the minimum open probability, p_min_, is p_min_ = 1/(1 + L_S4_^up^) (Figure 2C). The minimum and maximum open probabilities were converted into equilibrium constant for the C-O transitions with all S4s resting, L_S4_^up^, and all S4s activated, L_S4_^down^, respectively: L_S4_^up^ = (p_min_^−1^ − 1)^−1^ and L_S4_^down^ = (p_max_^−1^ − 1)^−1^. The free energy of the opening transition was calculated by ∆G_S4_^up^ = RT ln (L_S4_^up^) and ∆G_S4_^down^ = RT ln(L_S4_^down^). The changes in the free energy of opening due to S4 motion was calculated by ∆∆G = ∆G_S4_^down^ − ∆G_S4_^up^ = RT ln (L_S4_^down^/L_S4_^up^). The ratio of L_S4_^down^/L_S4_^up^ gives the coupling constant L, a measure of how much the movement of S4 alters the energetics of the opening transition. The changes in the free energy of coupling due to mutations were calculated by ∆∆G = RT ln (L_wt_/L_mut_).

### 4.6. Molecular Modeling

A morph video from gate closed to open was created in University of California San Francisco Chimera 1.13rc software using a previously developed model for spHCN channels in the closed and open states [42].

## 5. Conclusions

In summary, we show that HCN channels display a much smaller increase in the open probability when all S4s move to the activated state compared to Kv channels. We propose that this looser coupling of HCN channels is one of the factors that allows for the inverted voltage gating in HCN channels. We also propose that similar movements of the voltage sensor S4 and the S6 activation gate occur in both Kv and HCN channels, but the relative movements of S4 and S6 are opposite in the two channels.

## Data Availability

The data that support the findings of this study are available from the corresponding author on reasonable request.

## Supplementary Materials

The following supporting information can be downloaded at: https://www.mdpi.com/article/10.3390/ijms25084309/s1.
