# Supplementary material for: Loose Coupling between the Voltage Sensor and the Activation Gate in Mammalian HCN Channels Suggests a Gating Mechanism
Source: Int J Mol Sci. 2024 Apr 13;25(8):4309. doi: 10.3390/ijms25084309 (PMC11050684; doi:10.3390/ijms25084309)
Supplement: Supplementary file 1 [file ijms-25-04309-s001.zip › HCN Loose Coupling IJMS Supp- 03-07-2024.pdf]

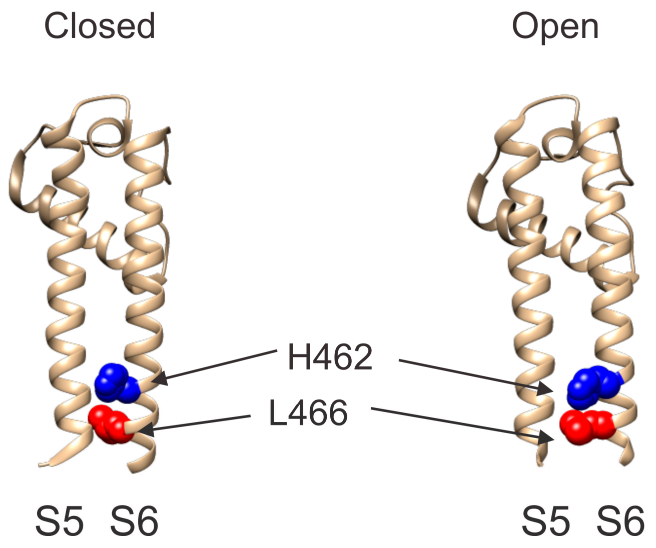

Figure. S1. HCN structures highlighting the interaction between H462 and L466
Left: HCN1 structure (PBD: 5U6O) with gate closed showing the homologous positions of H462 in blue and L466 in red from spHCN channels. Note that they point in slightly different directions (See Video. S1 for a better view). *Right:* HCN4 structure (PBD: 7NP3) with gate open showing the homologous positions of H462 in blue and L466 in red from spHCN channels. H462 and L466 are physically closer in the open state than in the closed state. Cd^{2+} binds to the two cysteines introduced at positions 462 and 466 and locks the channel in the open state. Only one S5 and S6 are shown for clarity.

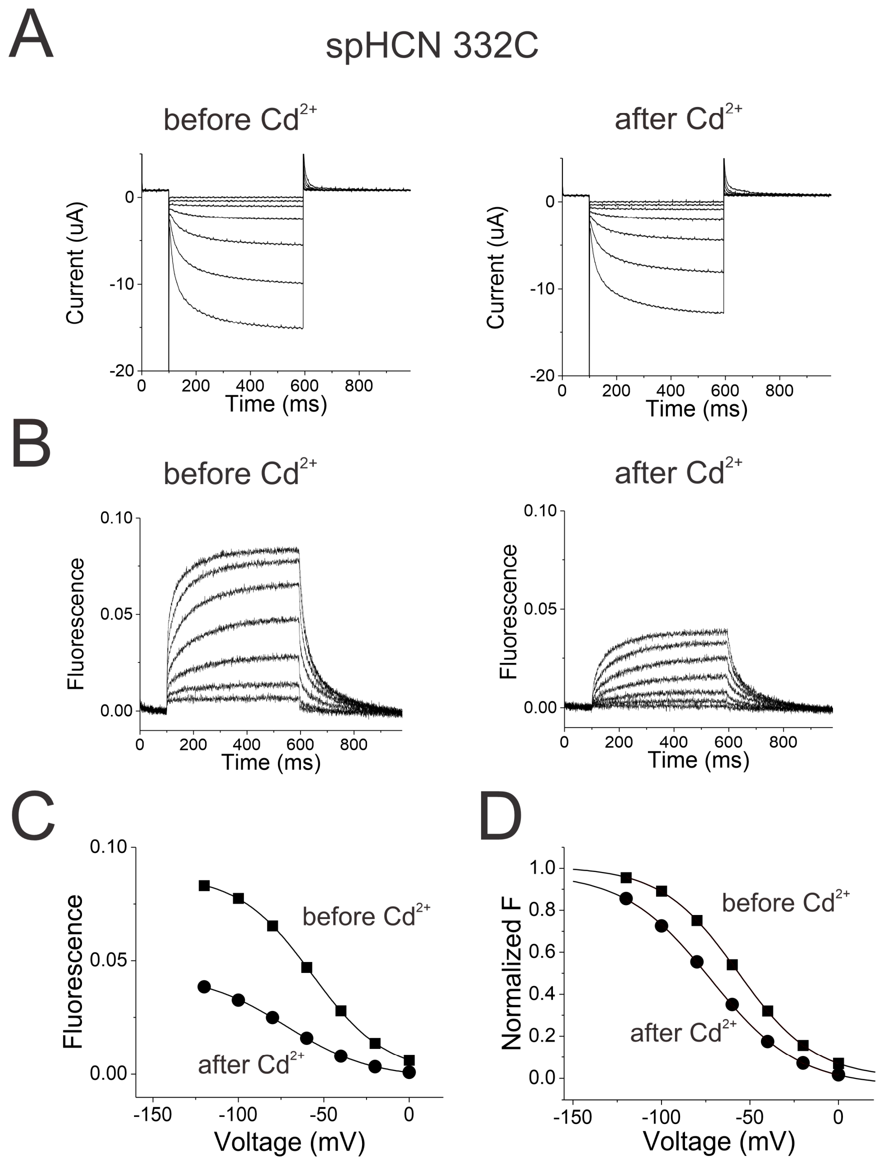

Figure. S2. Effects of Cd^{2+} application on spHCN 332C channels. (A) SpHCN 332C currents before (left) and after (right) injection of the oocyte with 50 nl of 10 µM $CdCl_2$ in response to voltage steps in Figure 1A. (**B)** SpHCN 332C fluorescence before (left) and after (right) injection of the oocyte with 50 nl of 10 µM $CdCl_2$ in response to voltage steps in Figure 1A. (**C)** FV relations before (squares) and after (circles) Cd^{2+} application on spHCN 332C channels. (**D)** Normalized FV relations from (**C**).

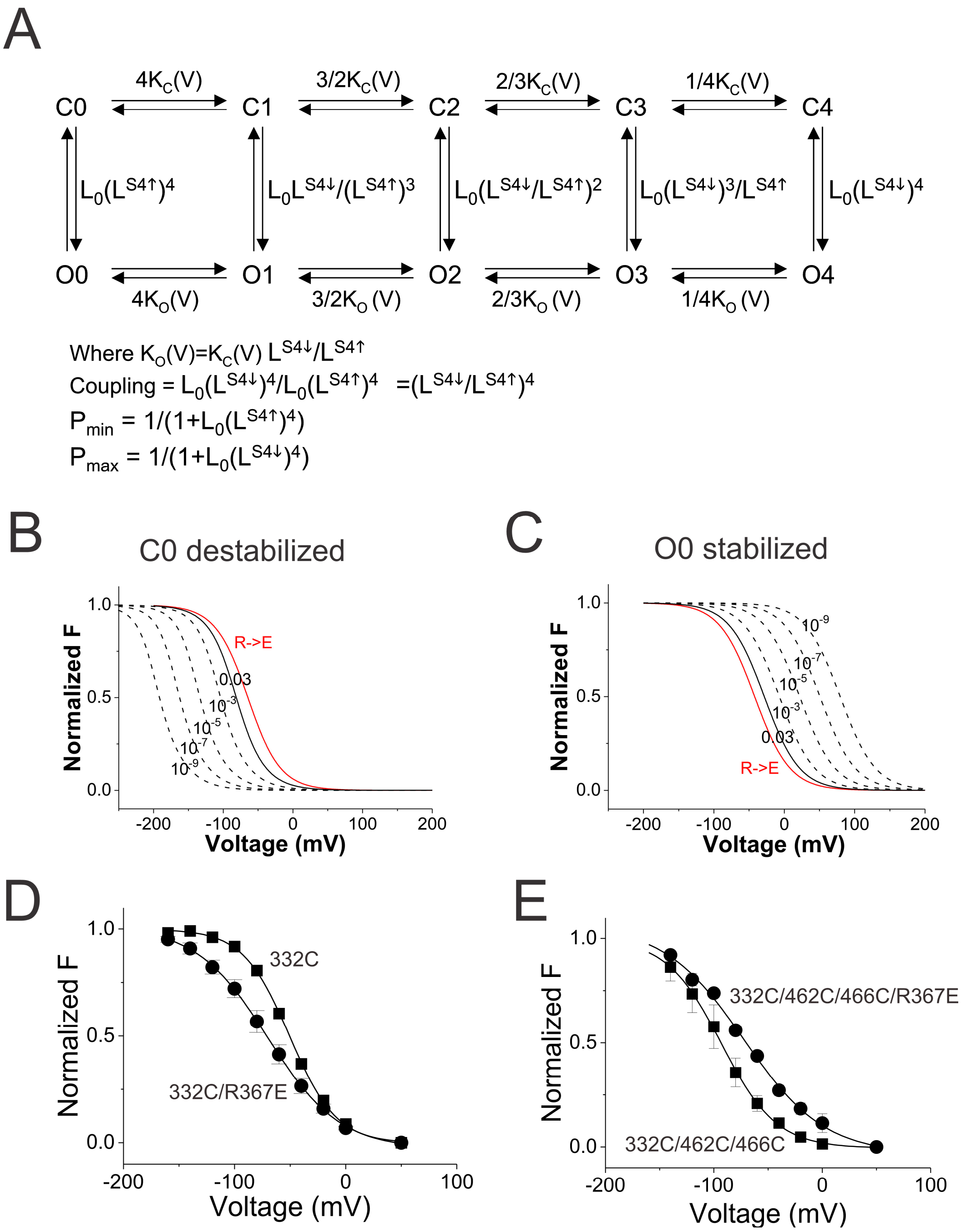

Figure. S3. Shifts in fluorescence caused by the R339E mutation are consistent with a loose coupling in HCN channels. (A) A 10-state model of HCN channels with 5 closed states (Ci, i= number of activated S4s) and 5 open states (Oi, i= number of activated S4s). The movements of the 4 S4s are assumed to be independent, and the opening transition is a concerted conformational change in all four subunits. (**B)** Predicted voltage dependence of S4 movements in the model from A for different p_{min} (10^{-3}– 10^{-9} in dashed lines, wt = 0.03 in black line, and R->E mutation = 0.43 in red line), where p_{min} was assumed to be altered by destabilization of the closed state C0.

Destabilizing the closed state was modeled by keeping K_O constant and calculating $K_C(V) = K_O(V)\ L^{S4\uparrow}/L^{S4\downarrow}$. **(C)** Predicted voltage dependence for the "locked-open" S4 movements in the model for different p_{min} (10^{-3}– 10^{-9} in dashed lines, wt = 0.03 in black line, and R->E mutation = 0.43 in red line), where p_{min} was assumed to be altered by the stabilization of the open state O0. Destabilizing the open state was modeled by keeping K_C constant and calculating $K_O(V) = K_C(V)\ L^{S4\downarrow}/L^{S4\uparrow}$. **(D)** FV relations of 332C (squares) and 332C/R367E (circles), from Figure. 5**C**. **(E)** "Locked-open" FV relation of 332C/462C/466C (squares) and 332C/R367E/462C/466C (circles), from Figure. 5**D**.

Video S1. Closing and opening conformational changes of S6 explain how Cd^{2+} interacting with H462C and L466C could lock the gate open. Molecular model of one S6 from our spHCN channel model in the closed and open states (See Methods). The sidechains at which we introduced the two cysteines (position H462 and L466) are shown in blue and red, respectively. In the closed state, H462 and L466 are not close enough to coordinate Cd^{2+} and point in different directions. In the open state, H462 and L466 are close enough so that Cd^{2+} could interact with both H462C and L466C simultaneously. Cd^{2+} could therefore lock the gate open by stabling the bend of the lower part of S6.
